# Efficacy and safety of PD-1 inhibitors in recurrent or metastatic nasopharyngeal carcinoma patients after failure of platinum-containing regimens: a systematic review and meta-analysis

**DOI:** 10.1186/s12885-023-11318-y

**Published:** 2023-11-30

**Authors:** Jian Luo, Wanying Xiao, Fengyang Hua, Yanqing Cao, Dongxia Wang, Xicheng Wang

**Affiliations:** 1 Department of Oncology, The First Affiliated Hospital of Guangdong Pharmaceutical University, Guangdong Pharmaceutical University, Guangzhou, 510062 China; 2 Affiliated Dongguan People’s Hospital, Southern Medical University (Dongguan People’s Hospital), Dongguan, 523058 China

**Keywords:** Recurrent or metastatic nasopharyngeal carcinoma, Platinum-containing regimens, PD-1, Efficacy, Safety

## Abstract

**Objective:**

There is a lack of standard salvage treatment options for recurrent or metastatic nasopharyngeal carcinoma (RM-NPC) that has failed platinum-containing regimens. Breakthroughs in immunotherapy have opened up new options for these patients. However, the efficacy and safety of immunotherapy have not been clarified. This study aimed to summarize and assess the efficacy and safety of PD-1 inhibitors in patients with RM-NPC who failed platinum-containing chemotherapy.

**Methods:**

Up to August 25, 2022, clinical trials of PD-1 inhibitors in RM-NPC patients who failed platinum-containing regimens were searched in the PubMed, Embase, Cochrane, and Web of Science databases. Retrieval subject terms included “nasopharyngeal carcinoma”, “metastatic”, “recurrence”, “PD-1”, and “PD-L1”. The clinical trials eligible for inclusion were systematically reviewed and meta-analyzed.

**Results:**

A total of 9 studies including 842 patients with RM-NPC were included in this meta-analysis. The results showed that PD-1 inhibitors had promising efficacy in patients with RM-NPC who failed platinum-containing regimens: objective response rate (ORR) was 24% (95% confidence interval [CI] 21–26%), disease control rate (DCR) was 52% (95% CI 45–58%), 1-year progression-free survival (PFS) rate was 25% (95% CI 18–32%), and 1-year overall survival (OS) rate was 53% (95% CI 37–68%). In terms of treatment-related adverse events (AEs), the incidence of grade ≥ 3 treatment-related AEs was 19% (95% CI 13–24%). In addition, we found that PD-1 inhibitors were more effective in patients with PD-L1 positive than in patients with PD-L1 negative nasopharyngeal carcinoma who had failed platinum-containing regimens (ORR 31% (95%CI 26–35%) vs. 21% (95% CI 17–25%)).

**Conclusion:**

PD-1 inhibitors may provide a survival benefit for patients with RM-NPC who have failed platinum-containing regimens and have the advantage of a good safety profile, making them a promising treatment option.

**Supplementary Information:**

The online version contains supplementary material available at 10.1186/s12885-023-11318-y.

## Introduction

Nasopharyngeal carcinoma (NPC) is a malignancy with marked geographic and racial differences in incidence and is prevalent in southern China, East Asia, and Southeast Asia [1, 2]. The International Agency for Research on Cancer statistics showed that about 133,000 new cases of NPC were diagnosed in 2020 [3]. For newly diagnosed early localized NPC, radiotherapy has a good effect. However, for locally advanced nasopharyngeal carcinoma (LA-NPC), induction chemotherapy and concurrent chemoradiotherapy are the first treatment options, but about 15–30% of patients will still develop recurrence or metastasis after initial treatment [4, 5].

In general, local treatment is not appropriate for patients with recurrent or metastatic nasopharyngeal carcinoma (RM-NPC), for whom the mainstream treatment plan remains palliative systemic chemotherapy with platinum-containing regimens [6, 7]. In 2016, a landmark phase III randomized controlled trial comparing the efficacy and adverse events of gemcitabine plus cisplatin (GP) and fluorouracil plus cisplatin (PF) in RM-NPC showed that the former had superior efficacy, median progression-free survival (PFS, 7.0 vs. 5.6 months), overall survival (OS, 29.1 vs. 20.9 months), and objective response rate (ORR, 64% vs. 42%) [8]. No serious adverse events occurred in both groups. From then on, the best first-line plan for RM-NPC was established.

There is no consensus on the next salvage treatment option for patients with RM-NPC who have failed platinum-containing chemotherapy regimens. As scientists continue to deepen their understanding of the immune system, it has made significant progress in the field of cancer treatment [9–11]. On the one hand, NPC has the characteristics of high expression of PD-1 and PD-L1 [12, 13]; on the other hand, there are a large number of infiltrating lymphocytes in NPC tissues [14], which provides a theoretical basis for the immunotherapy of NPC. In 2020, a multicenter phase II trial (CAPTAIN) reported the results of camrelizumab for RM-NPC after multiple chemotherapy failures: ORR of 28.2%, median PFS of 3.7 months, and median OS of 17.1 months [15]. In 2021, another clinical trial (POLARIS-02) reported the efficacy of toripalimab for the treatment of RM-NPC who have failed first-line platinum-containing regimens, with an ORR of 20.5%, median PFS of 1.9 months and median OS of 17.4 months [16]. Subsequently, several multi-center clinical trials of PD-1 inhibitors for the treatment of RM-NPC have been implemented worldwide. Initially, these researchers looked at the effectiveness of PD-1 inhibitors in NPC. However, the results reported by different studies were not completely consistent, and there was a lack of focused analysis of the adverse events caused by immunotherapy.

Therefore, we conducted this systematic review and meta-analysis to systematically summarize and compare the therapeutic effect and adverse events of various PD-1 inhibitors for the treatment of RM-NPC patients who failed platinum-containing regimens, and to compile more comprehensive data to provide important reference values for clinicians in developing individualized treatment plans for RM-NPC patients.

## Methods

This study is registered in the International Prospective Register of Systematic Reviews (PROSPERO) and the number is CRD42022373462. The conduct of this systematic review and meta-analysis adhered to PRISMA recommendations.

### Search strategy

Studies up to 25 August 2022 were searched from PubMed, Embase, Cochrane, and Web of Science databases. Retrieval subject terms included “nasopharyngeal carcinoma”, “metastatic”, “recurrence” “PD-1”, and “PD-L1”. We only included studies where the language of publication was English. More detailed literature searches and screening steps are described in Supplementary Text 1.

### Studies selection

Inclusion criteria: (1) Patients with pathologically diagnosed RM-NPC. (2) RM-NPC patients who failed platinum-containing regimens. (3) RM-NPC patients treated with PD-1 inhibitors alone. (4) Included studies were required to contain complete information on efficacy metrics and incidence of adverse events. Exclusion criteria include: (1) The patients also had a head and neck tumor other than NPC. (2) The treatment regimen includes drugs other than PD-1/PD-L1 inhibitors as combination therapy. (3) Article types of reviews, retrospective analyses, case reports, letters, editorials, and meta-analyses were also excluded.

### Data acquisition and quality assessment

Two researchers independently extracted the efficacy indicators and anti-PD-1 treatment-related adverse events data of patients with RM-NPC. Efficacy evaluation indexes included objective response rate (ORR: complete response (CR) + partial response (PR)), OS, PFS, and disease control rates (DCR: CR + PR + stable disease (SD)). Adverse events (AEs) data included the incidence of treatment-related AEs of any grade and grade ≥ 3. The detailed information extracted includes first author, year of publication, trial design, treatment and dose, PD-L1 positive, line of therapy, median age, 1-year PFS, 1-year OS, ORR, DCR, any AEs, and grade ≥ 3 AEs. Since most of our included studies were single-arm or uncontrolled studies, we used the Newcastle-Ottawa Scale (NOS) tool to evaluate the quality of the studies [17]. Only studies with a score of more than four stars were included in our subsequent analysis. Studies with more than four stars were included for further analysis. Any disagreements arising from data extraction and literature quality evaluation were resolved in consultation with the third investigator.

### Statistical analysis

Stata 14.0 software (Stata Corporation, College Station, Texas, UAS) was used to perform the statistical analysis. The chi-square test and I^2^ statistics were used to measure heterogeneity. The fixed effect model is used if *P* > 0.1 or I^2^ < 50% of the heterogeneity. On the other hand, the random effect model will be used if the heterogeneity is clear. Publication bias was evaluated using Egger’s test.

## Results

### Eligible studies and characteristics

**Fig. 1:**
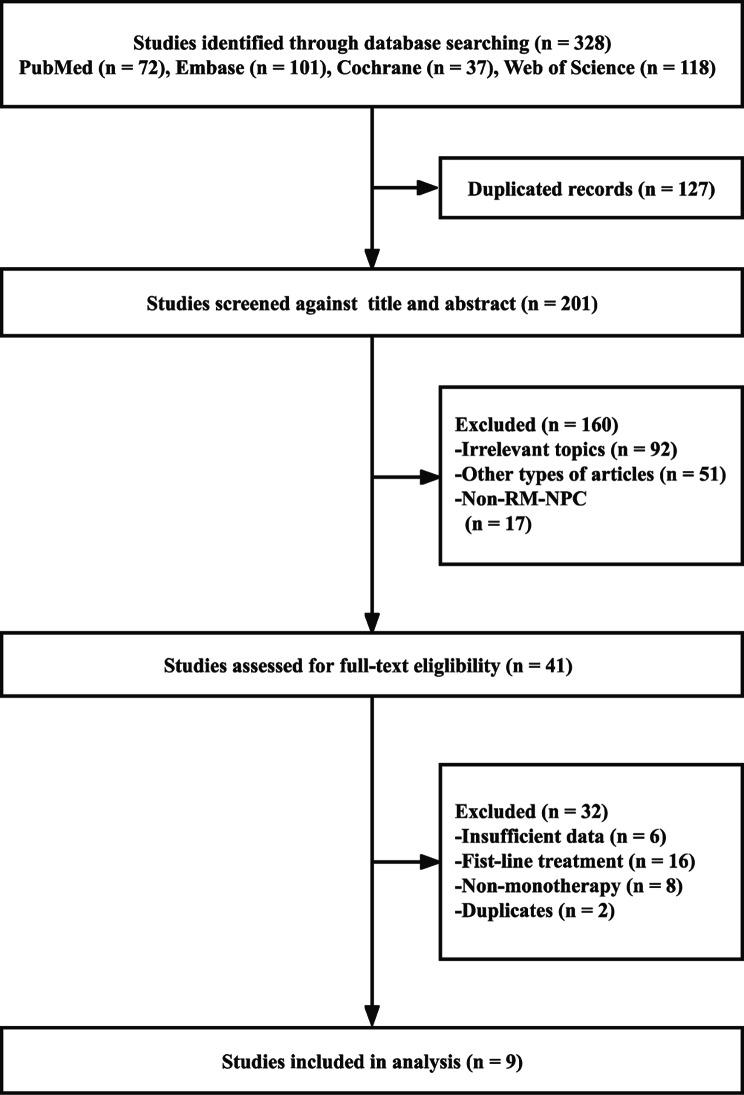
Flow chart of the screening process in the meta-analysis

We obtained a total of 328 articles using the above-mentioned search subject phrases from the PubMed, Embase, Cochrane, and Web of Science databases; 201 articles remained after excluding duplicates. Next, 160 articles were further excluded by reading the titles and abstracts, leaving 41 articles for full-text reading. At last, just 9 articles remained which met the inclusion criteria were obtained for meta-analysis. The detailed process of literature screening is shown in Fig. 1.

Finally, 842 patients with RM-NPC after the failure of platinum-containing regimens from 9 studies were included in this meta-analysis. Seven articles are single-arm studies [15, 16, 18–22], including two phase I studies, four phase II studies, and one phase I/II study. Two were randomized controlled studies [23, 24], including one Phase II study and one Phase III study. Anti-PD-1 agents include pembrolizumab, nivolumab, camrelizumab, toripalimab, penpulimab, and spartalizumab. Six studies reported the rate of positive PD-L1 expression, and three of them compared the difference in efficacy of PD-1 inhibitors in NPC patients with positive versus negative PD-L1 expression [15, 16, 25], showing that anti-PD-1 therapy could benefit regardless of PD-L1 expression status, and the benefit was more pronounced in those with positive PD-L1 expression (ORR, 31% vs. 21%). The results of the literature quality assessment showed that 8 of the 9 studies were assessed at 7–9 stars, and the remaining one was assessed at 5 stars. The detailed characteristics of the final nine included studies are described in Table 1.

**Table 1:** The detailed characteristics of the final nine included studies

First author	Year	Trial design	Treatment	Typle	PD-L1positive(%)	Line of therapy ≥ 2(%)	Patients enrolled	Dose	Median age	Median OS (months, 95% CI)	Median PFS (months, 95% CI)	1-Year OS(%)	1-Year PFS(%)	ORR (%)	DCR (%)	Any AEs	Grade ≥ 3 AEs	NOS score
C. Hsu	2017	Single-arm phaseI	Pembrolizumab	PD-1	NA	81.5	27	10mg/kg q2w	52	16.5 (10.1–NR)	6.5(3.6–13.4)	63	33.4	25.9	77.8	74.1	29.6	7
Brigette B.Y. Ma	2018	Single-arm phaseII	Nivolumab	PD-1	40	100	44	3mg/kg q2w	57	17.1(10.9-NR)	2.8(1.8–7.4)	59	19.3	20.5	54.5	NA	22.2	8
Wenfeng Fang	2018	Single-arm phaseI	Camrelizumab	PD-1	NA	76	91	1 mg/kg, 3 mg/kg, and 10 mg/kg, and a bridging dose of 200 mg per dose once q2w	45	NA	5.6(3.3–7.9)	NA	NA	34	59	97	17	8
Yunpeng Yang	2021	Single-arm phaseII	Camrelizumab	PD-1	73.1	100	156	200mg q2w	48	17.4(15.2–21.9)	3.7(2.0-4.1)	NA	NA	28.2	54.5	99.4	33.3	8
Feng-Hua Wang	2021	Single-arm phaseII	Toripalimab	PD-1	25.3	48.4	190	3mg/kg q2w	46	17.4(11.7–22.9)	1.9(1.8–3.5)	NA	NA	20.5	40	74.2	14.2	8
Delord, J. P.	2017	Single-arm phase I/II	Nivolumab	PD-1	NA	NA	24	240mg q2w	NA	NR	2.4(1.5-NR)	NA	NA	20.8	45.8	NA	NA	5
Xiaozhong Chen	2020	Single-arm phase II	Penpulimab	PD-1	38.7	100	111	200mg q2w	NA	NA	NA	NA	NA	27	49.5	79.2	14.6	7
Caroline Even	2021	Randomized Phase II	Spartalizumab vs.Chemotherapy	PD-1	95.1	80.5	82	400mg q4w	51	25.2(13.1-NR)	1.9(1.8–3.6)	NA	27.1	17.1	42.7	72	17.1	9
A.T. Chan	2021	Randomized Phase III	Pembrolizumab vs.Chemotherapy	PD-1	74.4	NA	117	200mg q3w	NA	17.2(11.7–22.9)	4.1(2.1–5.6)	40.2	NA	21.4	50.4	61.2	10.3	7

PFS: Progression-free survival; OS: Overall survival; ORR: Objective response rate; DCR: Disease control rate; NA: Not available; NR: Not reach; AEs: Adverse events

### Efficacy

**Fig. 2:**
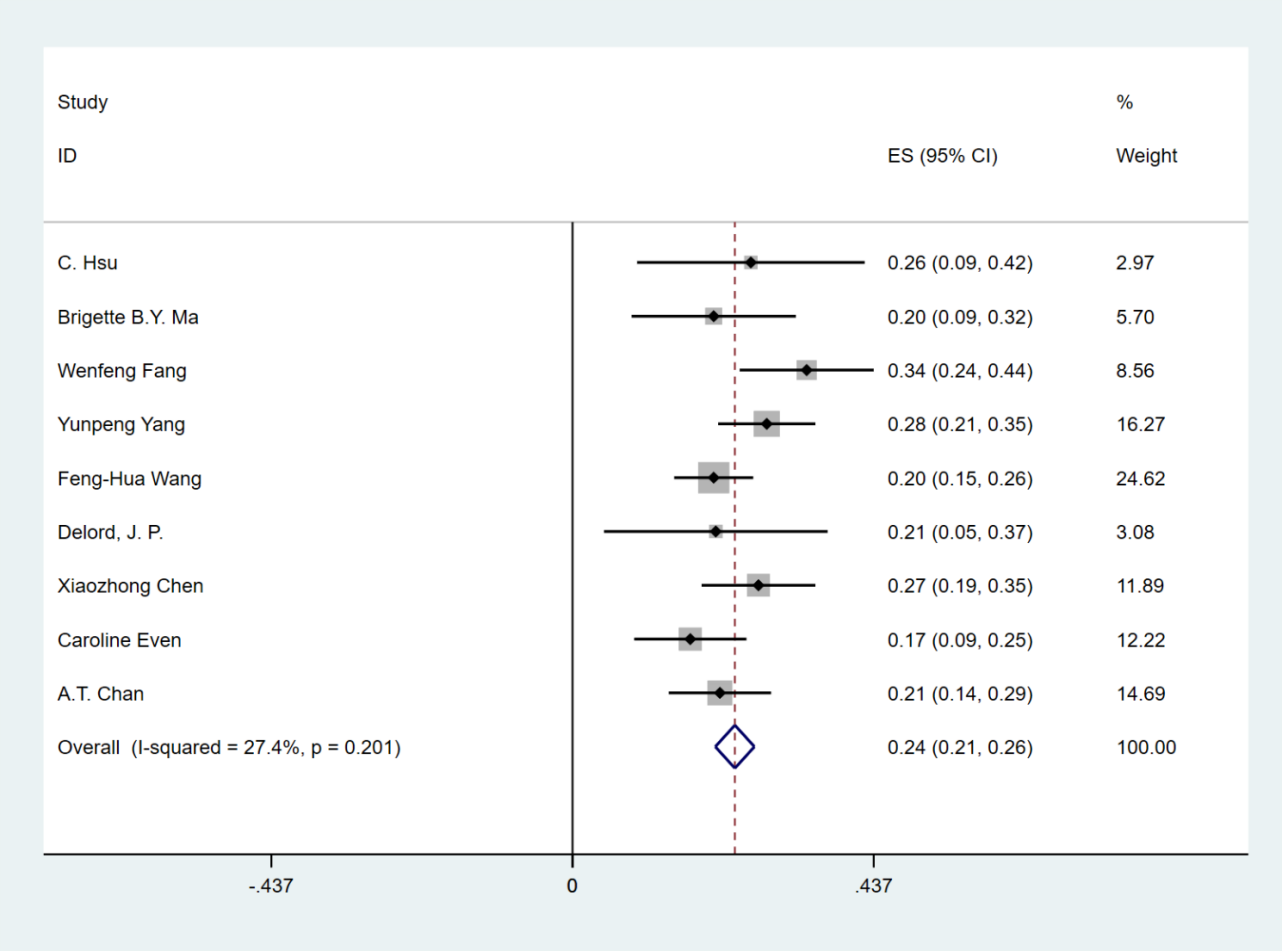
Forest plots of objective response rate (ORR) in RM-NPC patients with platinum-containing regimen failure. ES: effect size; CI: confidence interval

**Fig. 3:**
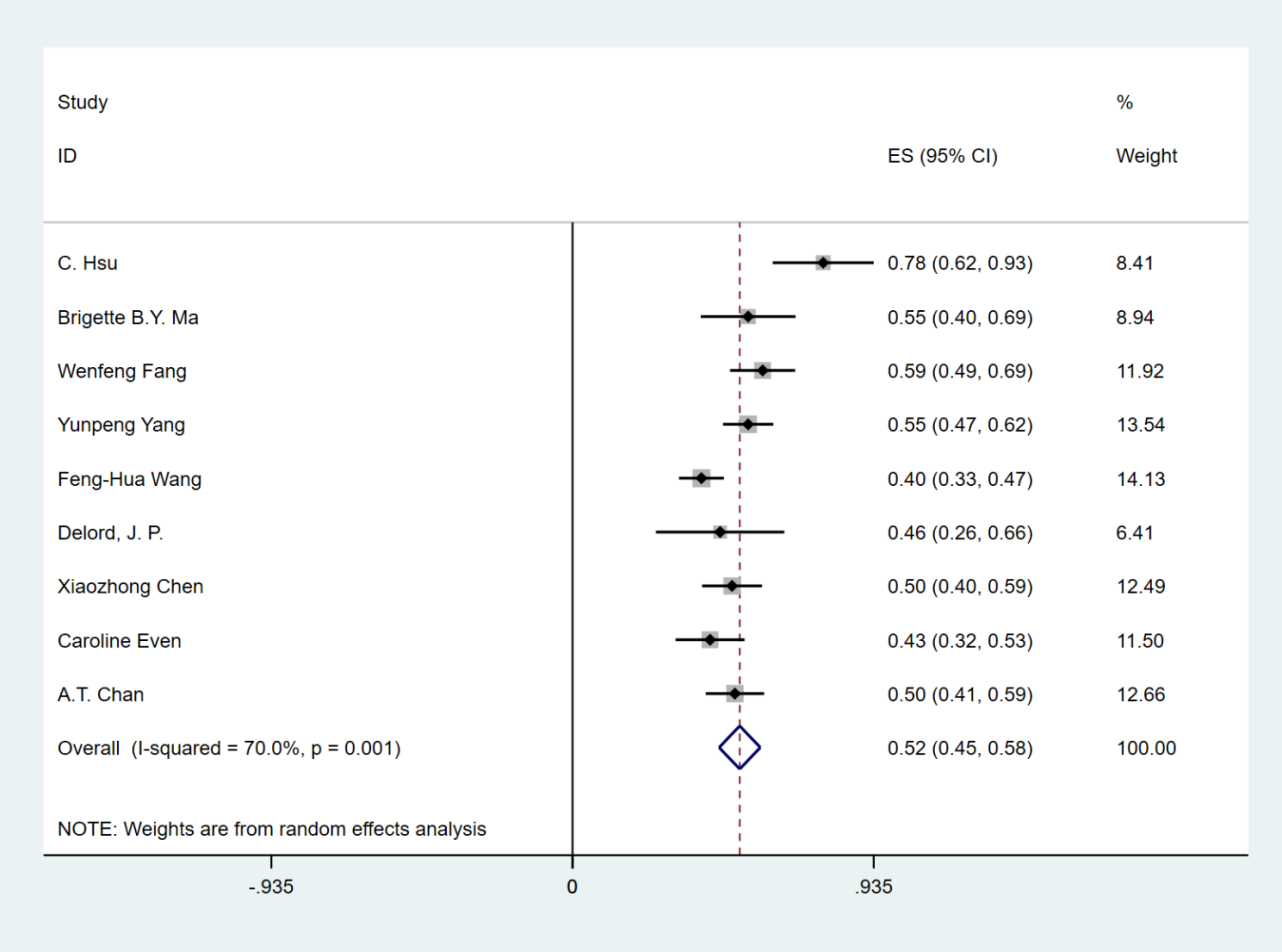
Forest plots of disease control rates (DCR) in RM-NPC patients with platinum-containing regimen failure

All 842 patients in the study reported ORR and DCR, with the range of ORR from 17.1 to 28.2% and DCR from 40 to 77.8%. PD-1 inhibitor in the treatment of patients with RM-NPC who failed platinum-containing regimen, the ORR was 24% (95% CI 21–26%), (I^2^ = 27.4%, *P* = 0.201) fixed effect model was used (Fig. 2), DCR was 52% (95% CI 45–58%), (I^2^ = 70%, *P* = 0.001), and random effect model was used (Fig. 3). This has achieved efficacy similar to that of single-agent chemotherapy (ORR:23.5%) [26].

**Fig. 4:**
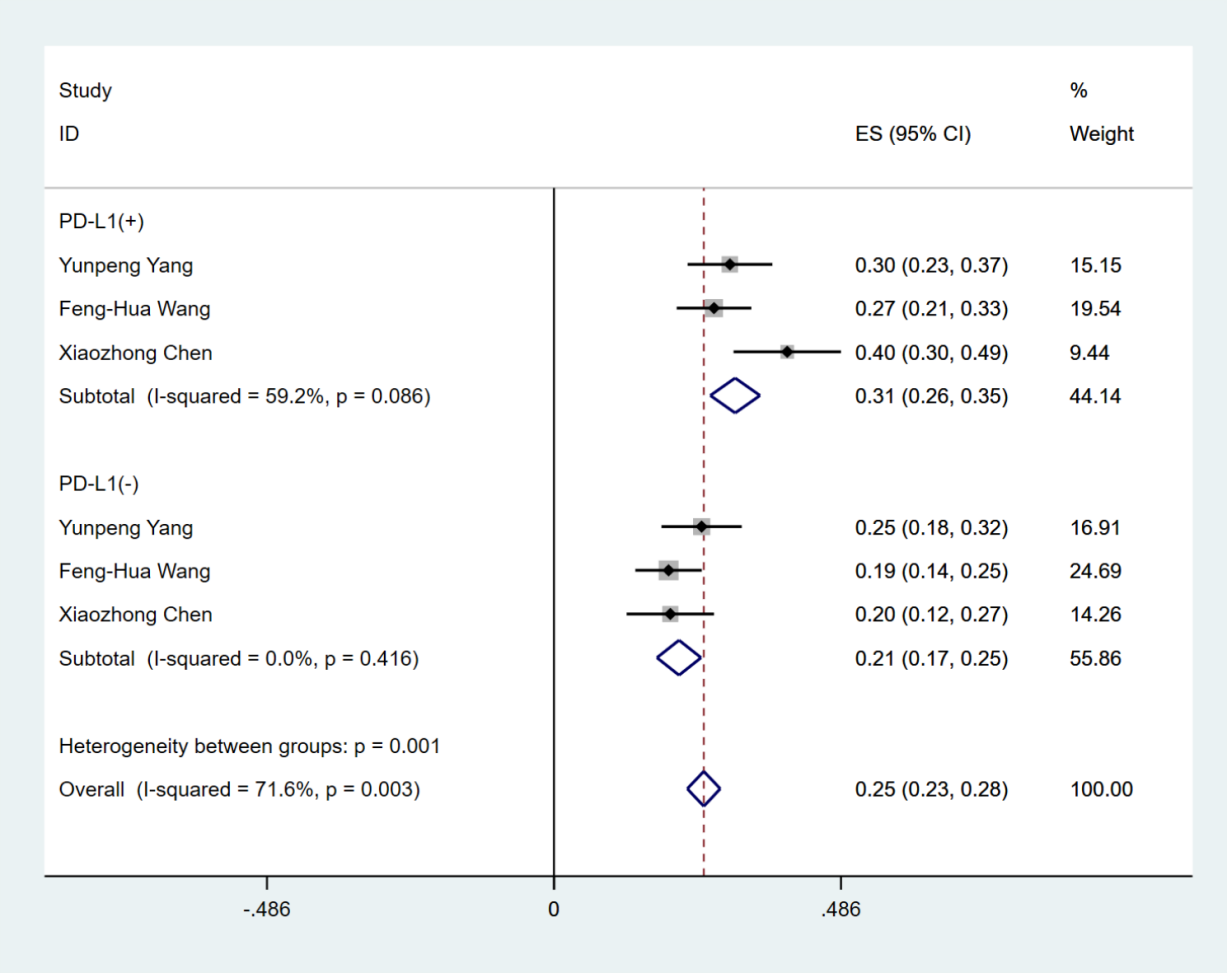
The ORR of PD-L1 expression in RM-NPC patients with platinum-containing regimen failure

We evaluated the expression status of PD- L1 in tumor tissues of a total of 441 NPC patients in three studies, of which 205 were PD-L1 positive and 236 were PD-L1 negative. The ORR for PD-1 inhibitors used to treat PD-L1-positive RM-NPC patients was 31% (95%CI 26–35%), (I^2^ = 59.2%, *P* = 0.086), while the ORR for PD-L1-negative patients was 21% (95% CI 17–25%), (I^2^ = 0.00%, *P* = 0.416) (Fig. 4). These results suggest that PD-1 inhibitors used in PD-L1-positive RM-NPC patients have better ORR than PD-L1-negative patients.

**Fig. 5:**
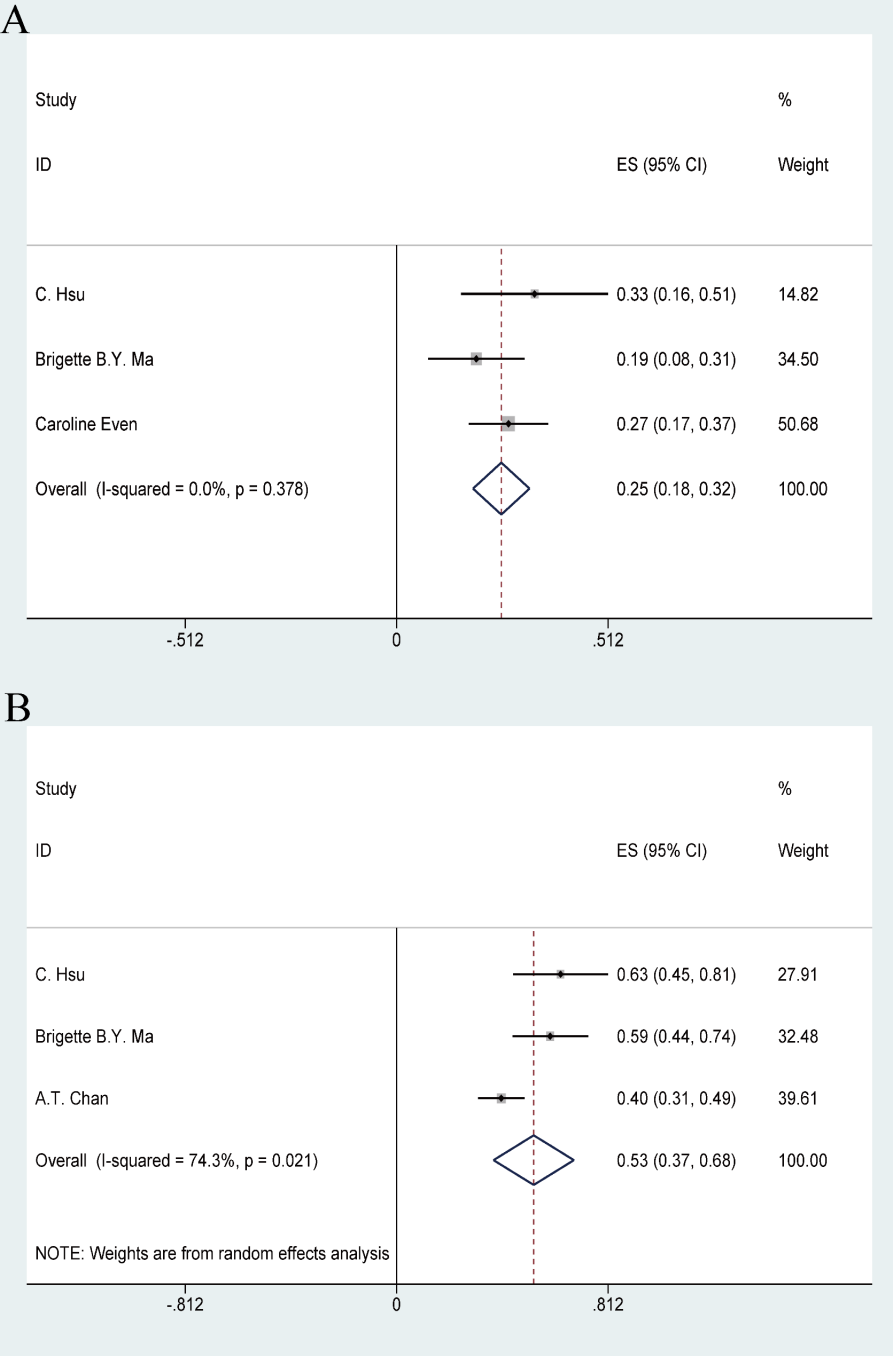
(**A**) Forest plots of 1-year progression-free survival (PFS) rate in RM-NPC patients with the platinum-containing regimen failure. (**B**) Forest plots of 1 1-year overall survival (OS) rate in RM-NPC patients with the platinum-containing regimen failure

Three studies with 126 patients reported 1-year PFS ranging from 19.3 to 33.4%. 1-year PFS was 25% (95% CI 18–32%), (I^2^ = 0.0%, *P* = 0.378) using a fixed effect model (Fig. 5A). A total of 161 patients in three studies reported 1-year OS, ranging from 40.2 to 63%. 1-year OS was 53% (95% CI 37–68%), (I^2^ = 74.3%, *P* = 0.021) using a random effect model (Fig. 5B).

### Safety

**Fig. 6:**
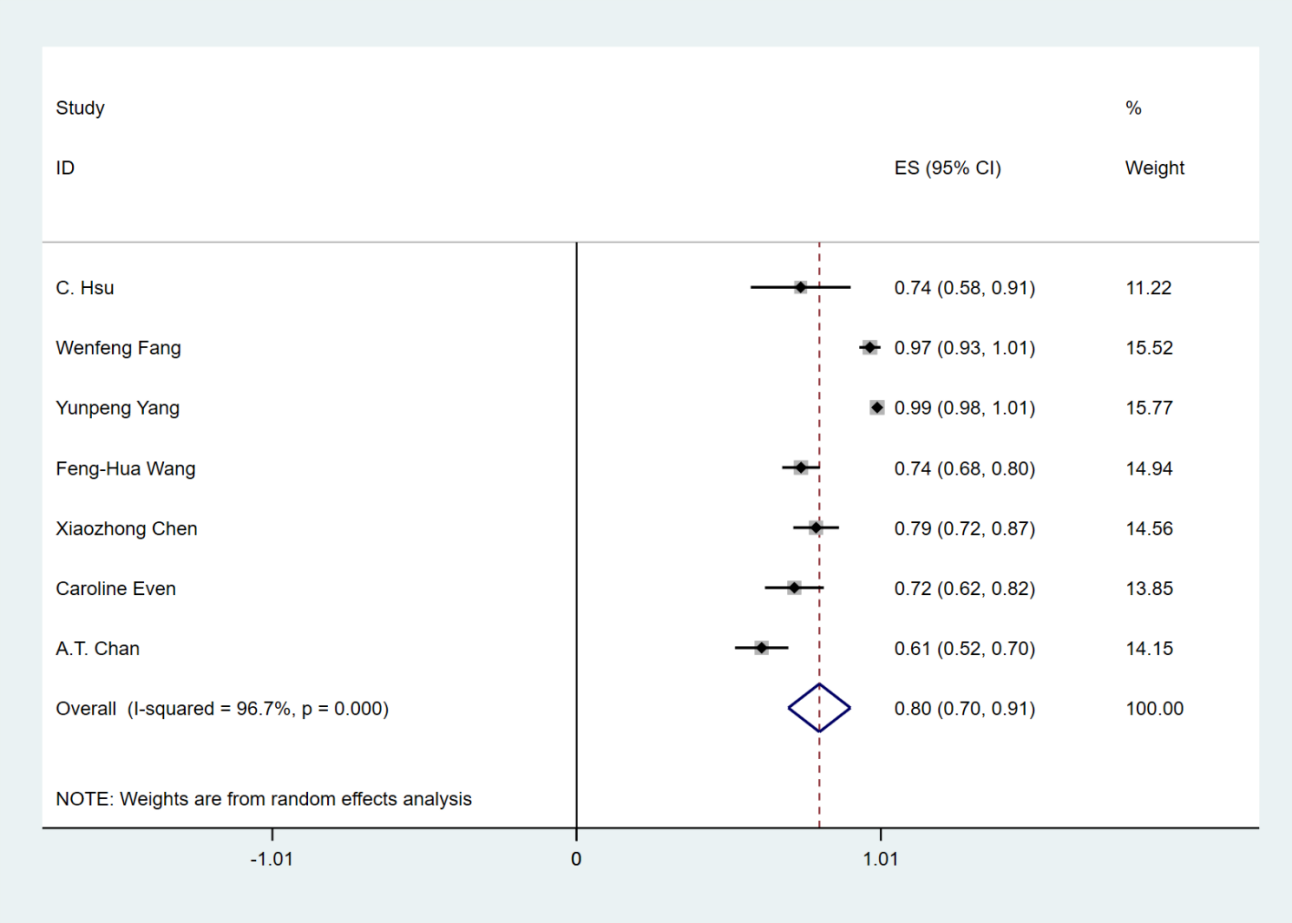
Forest plots of the incidence of treatment-related any-grade adverse events in RM-NPC patients with platinum-containing regimen failure

**Fig. 7:**
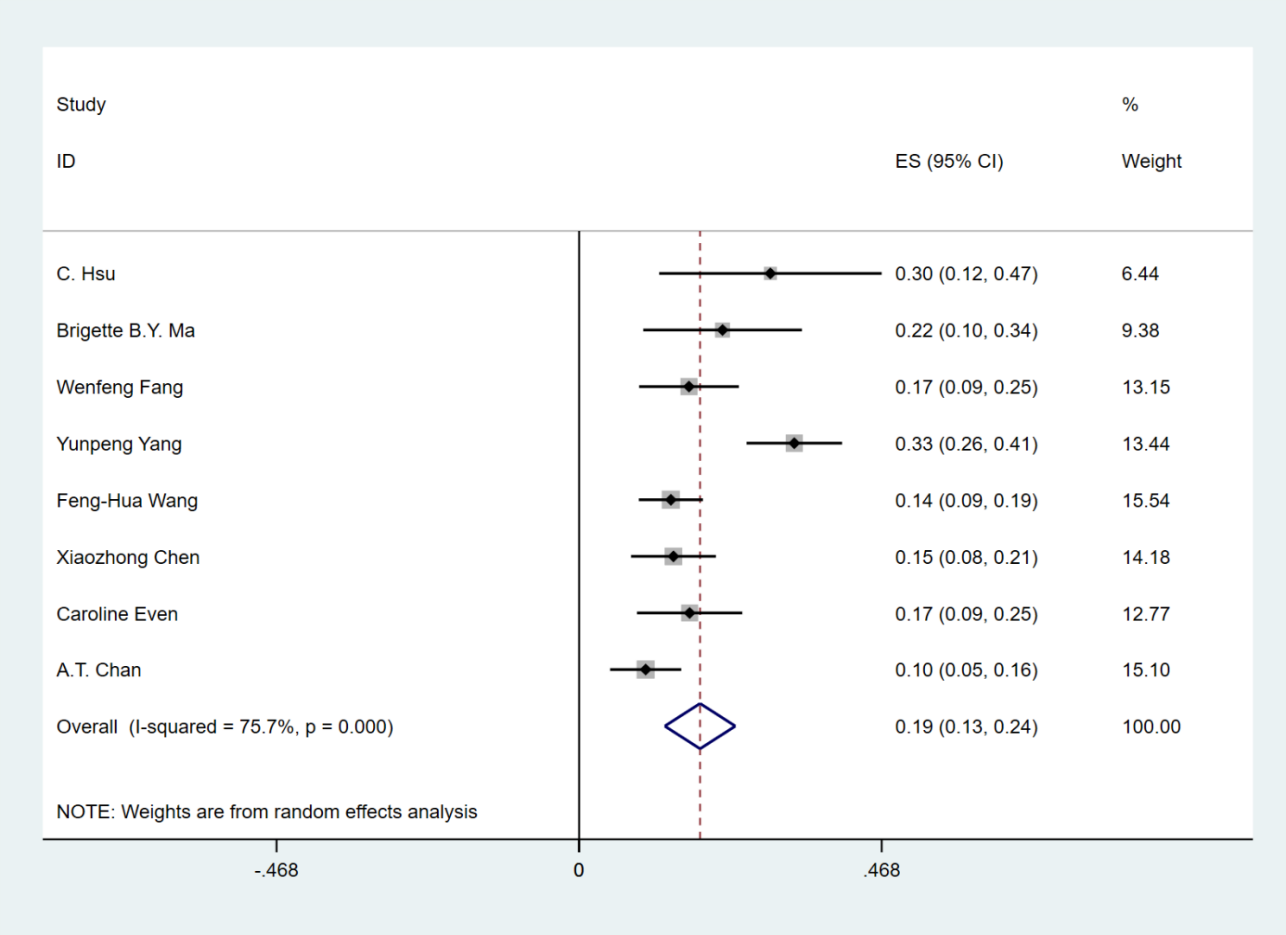
Forest plots of the incidence of treatment-related grade ≥ 3 adverse events in RM-NPC patients with platinum-containing regimen failure

**Fig. 8:**
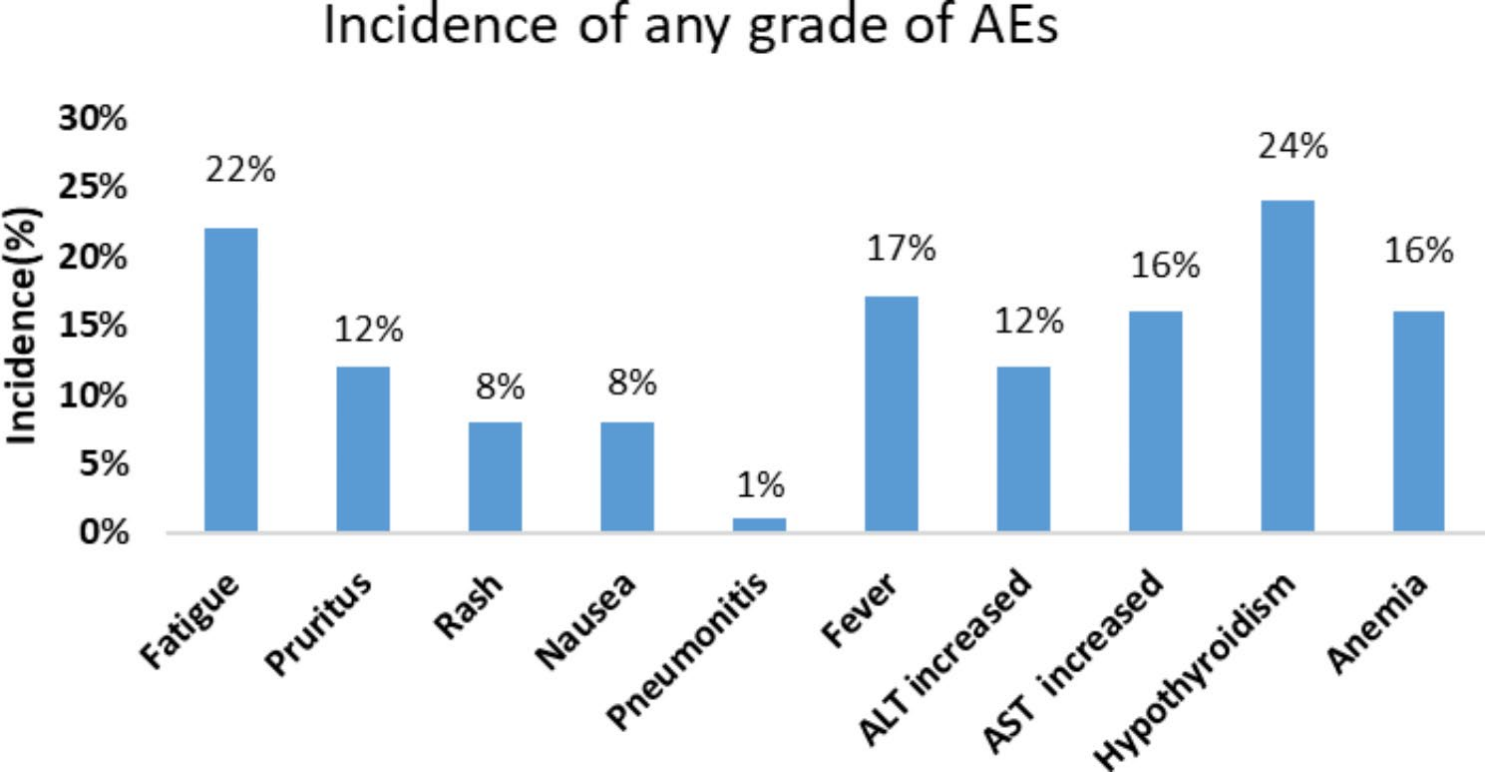
The incidence of any grade of treatment-related adverse events in RM-NPC patients with platinum-containing regimen failure. ALT: alanine aminotransferase; AST: aspartate aminotransferase

Seven studies with 774 patients reported any grade of treatment-related AEs. The incidence of any grade of treatment-related AEs was 80% (95%CI 70–91%), (I^2^ = 96.7%, P = 0.000), and the random effect model was used (Fig. 6). Eight studies reported treatment-related AEs with grade 3 or higher. The incidence of grade 3 or higher treatment-related AEs was 19% (95%CI 13–24%), (I^2^ = 75.7%, P = 0.000), and the random effect model was used (Fig. 7). The incidence of any grade of treatment-related AEs is shown in Fig. 8: hypothyroidism 24%, fatigue 22%, fever 17%, anemia 16%, AST increased 16%, ALT increased 12%, pruritus 12%, rash 8%, nausea 8%, pneumonitis 1%. From this result, we can see that PD-1 inhibitor monotherapy used to treat RM-NPC patients who have failed treatment with platinum-containing regimens still has a relatively high overall rate of treatment-related adverse events, but the rate of grade 3 or higher treatment-related adverse events is only 19%, which is much lower than the rate of 34.4% for single-agent chemotherapy [27].

### Publication bias

We assessed the publication bias of PD-1 inhibitors in R/M NPC patients after failure of platinum-containing regimens by Egger’s test of *P* > 0.01 and found that there was no significant publication bias in the relevant results, as shown in Table 2.

**Table 2:** Results of publication bias

Effect size	Egger’s test *(P*)
ORR	0.696
DCR	0.204
PFS	0.733
OS	0.121
Any grade of AEs	0.048
Grade ≥ 3 AEs	0.171

ORR: Objective response rate; DCR: Disease control rate; PFS: Progression-free survival; OS: Overall survival; AEs: Adverse events

## Discussion

With the increasing understanding of the immune system by scientists, immunotherapy is becoming a new therapy for many tumors, especially in the treatment of patients with advanced tumors that are not suitable for radical surgical resection treatment [28]. Immunotherapy for tumors includes tumor vaccines, adoptive immune cell therapy, immunomodulators, and immune checkpoint inhibitors [29–31]. Currently, PD-1 inhibitors are the most commonly used immunotherapies in treating NPC. With the publication of increasing clinical trial results and the formation of evidence-based medical evidence, the level of evidence of PD-1/PD-L1 inhibitors in the treatment of NPC is expected to be further improved in the future.

For patients with RM-NPC, the National Comprehensive Cancer Network guidelines recommended gemcitabine plus cisplatin (class 1 recommendation) as the first-line systemic treatment of choice. With the breakthroughs in immunotherapy, some studies have found that the efficacy of immunotherapy combined with chemotherapy is superior to chemotherapy alone in local RM-NPC. A phase III clinical trial (CAPTAIN-1) reported the efficacy of camrelizumab plus gemcitabine and cisplatin versus placebo plus gemcitabine and cisplatin, and the results suggested that the camrelizumab group was significantly more effective than the placebo control group: median PFS (10.8 vs. 6.9 months), DOR (9.9 vs. 5.7 months), ORR (88.1% vs. 80.6%), and both regimens were free of major adverse events [32]. Another phase III clinical trial found that toripalimab plus gemcitabine and cisplatin also had superior efficacy compared to placebo in combination with gemcitabine and cisplatin: median PFS (11.7 vs. 8.0 months), DOR (10.0 vs. 5.7 months), ORR (77.4% vs. 66.4%) [33]. These findings suggest that PD-1 inhibitors in combination with chemotherapy have superior efficacy and fewer adverse events than chemotherapy alone in local RM-NPC, making them more suitable as first-line standard treatment options.

For patients with RM-NPC who had failed treatment with a platinum-containing regimen, monotherapy was previously mainly performed with a new chemotherapeutic agent not used in the first-line regimen, including docetaxel, capecitabine, and gemcitabine [26, 27, 34]. With the development and wide application of immunotherapy, in recent years, some studies have tried to explore the efficacy and safety of immunotherapy for RM-NPC after the failure of platinum-containing regimens. A phase II clinical study (NCT03605967) comparing the efficacy of Spartalizumab and chemotherapy in RM-NPC patients who failed platinum-containing regimens showed a better Median OS (25.2 vs. 15.5 months) but worse Median PFS (1.9 vs. 6.6 months) and ORR (17.1% vs. 35.0%), Spartalizumab has a good safety profile like other PD-1 inhibitors [23]. Another phase III clinical trial (KEYNOTE-122) compared the efficacy and adverse events of pembrolizumab (n = 117) with standard single-agent chemotherapy (n = 116) and showed a median OS of 17.2 vs. 15.3 months, median PFS was (4.1 vs. 5.5 months), ORR was (21.4% vs. 23.3%), and the incidence of grade 3–5 adverse events between the two groups was (10.3% vs. 43.8%) [24]. These findings suggest that in patients with RM-NPC who have failed platinum-containing regimens, PD-1 inhibitors can achieve similar efficacy to single-agent chemotherapy, but the incidence of adverse reactions is significantly lower than chemotherapy, and the compliance and tolerance of patients are better than chemotherapy regiments.

Our meta-analysis also showed similar results as above: ORR 24% (95% confidence interval [CI] 21–26%), DCR 52% (95% CI 45–58%), 1-year PFS rate 25% (95% CI 18–32%), and 1-year OS rate 53% (95% CI 37–68%). The incidence of grade ≥ 3 treatment-related AEs were 19% (95% CI 13–24%), and the incidence of treatment-related any grade AEs was 80% (95% CI 70–91%). Among all AEs related to PD-1 inhibitor treatment, the highest incidence rate was 23% for hyperthyroidism and 22% for fatigue, followed by 17% for fever, 16% for anemia, 16% for AST increased, and finally, 12% for ALT increased, 12% for pruritus, 8% for rash, 8% for nausea, and 1% for pneumonia. Interestingly, a previous meta-analysis reported the efficacy and safety of PD-1/PD-L1 in the treatment of RM-NPC, and the results were similar to those in this study (ORR 25%, and DCR 60%) [35]. The difference with our study is that only 3 clinical trials were included in this study, and the subjects included in this study were different from our study. The subjects in this study were patients with RM-NPC, while the subjects in our study were patients with RM-NPC who failed the platinum-containing regimen. Overall, these findings suggest that PD-1 inhibitors in the treatment of patients with RM-NPC who have failed platinum-based regimens have similar efficacy as single-agent chemotherapy, but the incidence of adverse events is significantly lower than chemotherapy. Therefore, immunotherapy may be a promising approach for patients with RM-NPC who have failed platinum-containing regimens.

Programmed cell death-Ligand 1(PD-L1) is overexpressed in many types of tumor cells and is strongly associated with a patient’s prognosis. A meta-analysis by Huang ZL et al. compared the relationship between different PD-L1 expression statuses and the prognosis of patients with NPC and found no statistical difference between positive or negative PD-L1 expression in tumor tissues and the prognosis of NPC. However, in a subgroup analysis, it was found that those with positive PD-L1 expression in immune cells of NPC patients had a better prognosis, and the higher the expression level, the longer the OS [36]. To clarify whether the efficacy of PD-1 inhibitors treatment for RM-NPC is related to the PD-L1 expression status of the tumor, we compared the ORR of PD-1 inhibitors in patients with PD-L1-positive and PD-L1-negative expressing NPC and showed that PD-1 inhibitors can benefit regardless of PD-L1 expression status, and the benefit was more pronounced in those with positive PD-L1 expression (ORR, 31% vs. 21%). Accordingly, we speculate that PD-L1 expression status may correlate with PD-1 inhibitors efficacy, with better efficacy in those with positive PD-L1 expression, but more studies are needed to further confirm this conclusion.

Admittedly, this study has some limitations. On the one hand, most of the eligible studies included were single-arm clinical trials, some of which had small sample sizes, which may influence conclusions; on the other hand, there were differences in the systematic treatment received by these patients before treatment with PD-1 inhibitors, which may affect the overall survival of patients and lead to a large potential heterogeneity between studies.

## Conclusion

The efficacy of PD-1 inhibitors for RM-NPC patients who failed treatment with platinum-containing regimens is similar to that of single-agent re-chemotherapy reported in previous studies, but PD-1 inhibitors have the advantages of fewer adverse effects and better tolerability. Of course, more large clinical studies are still needed to further confirm the above conclusions.

### Electronic supplementary material

Below is the link to the electronic supplementary material.

Supplementary Material 1

## Data Availability

The authors declare that the data covered in this study can be found in the Supplementary file or requested from the corresponding author upon reasonable request.

## Abbreviations

AEs: Adverse events
ALT: Alanine aminotransferase
AST: Aspartate aminotransferase
CI: Confidence interval
DCR: Disease control rate
ES: Effect size
GP: Gemcitabine plus cisplatin
LA-NPC: Locally advanced nasopharyngeal carcinoma
NA: Not available
NPC: Nasopharyngeal carcinoma
NR: Not reach
ORR: Objective response rate
OS: Overall survival
PD-1: Programmed death-ligand 1
PF: Fluorouracil plus cisplatin
PFS: Progression-free survival
RM-NPC: Recurrent or metastatic nasopharyngeal carcinoma
