# Supplementary material for: Efficacy and safety of PD-1 inhibitors in recurrent or metastatic nasopharyngeal carcinoma patients after failure of platinum-containing regimens: a systematic review and meta-analysis
Source: BMC Cancer. 2023 Nov 30;23:1172. doi: 10.1186/s12885-023-11318-y (PMC10688056; doi:10.1186/s12885-023-11318-y)
Supplement: Supplementary file 1 — Supplementary Material 1 [file 12885_2023_11318_MOESM1_ESM.docx]

**Supplementary Text 1. Detailed search terms and steps in all databases**

**Pubmed：**

((((((((((((((((PD-1[Title/Abstract]) OR (PD-L1[Title/Abstract])) OR (pembrolizumab[Title/Abstract])) OR (nivolumab[Title/Abstract])) OR (dostarlimab[Title/Abstract])) OR (atezolizumab[Title/Abstract])) OR (avelumab[Title/Abstract])) OR (durvalumab[Title/Abstract])) OR (tislelizumab[Title/Abstract])) OR (camrelizumab[Title/Abstract])) OR (cemiplimab[Title/Abstract])) OR (spartalizumab[Title/Abstract])) OR (sintilimab[Title/Abstract])) OR (sugemalimab[Title/Abstract])) OR (penpulimab[Title/Abstract])) AND ((((((metastatic[Title/Abstract]) OR (Neoplasm Metastasis[Title/Abstract])) OR (Neoplasm Metastases[Title/Abstract])) OR (Recurrence[Title/Abstract])) OR (Neoplasm Recurrence[Title/Abstract])) OR (Local Neoplasm Recurrence[Title/Abstract]))) AND (((((((((Nasopharyngeal neoplasms) OR (nasopharyngeal carcinoma[Title/Abstract])) OR (nasopharyngeal cancer[Title/Abstract])) OR (nasopharyngeal tumor[Title/Abstract])) OR (nasopharynx tumor[Title/Abstract])) OR (nasopharynx cancer[Title/Abstract])) OR (nasopharynx carcinoma[Title/Abstract])) OR (nasopharynx neoplasms[Title/Abstract])) OR (NPC[Title/Abstract]))

**Embase：**

#1 'nasopharyngeal neoplasms':ab,ti OR 'nasopharyngeal cancer':ab,ti OR 'nasopharyngeal tumor':ab,ti OR 'nasopharynx tumor':ab,ti OR 'nasopharynx cancer':ab,ti OR 'nasopharynx carcinoma':ab,ti OR 'nasopharynx neoplasms':ab,ti OR npc:ab,ti

#2 ‘metastatic’:ab,ti OR 'neoplasm metastasis':ab,ti OR 'neoplasm metastases':ab,ti OR recurrence:ab,ti OR 'neoplasm recurrence':ab,ti OR 'local neoplasm recurrence':ab,ti

#3 'PD-1':ab,ti OR 'PD-L1':ab,ti OR pembrolizumab:ab,ti OR nivolumab:ab,ti OR dostarlimab:ab,ti OR atezolizumab:ab,ti OR avelumab:ab,ti OR durvalumab:ab,ti OR toripalimab:ab,ti OR tislelizumab:ab,ti OR camrelizumab:ab,ti OR cemiplimab:ab,ti OR spartalizumab:ab,ti OR sintilimab:ab,ti OR sugemalimab:ab,ti OR penpulimab:ab,ti

#1 AND #2 AND #3

**Web of science：**

#1 ((((((((AB=(Nasopharyngeal neoplasms)) OR AB=(nasopharyngeal carcinoma)) OR AB=(nasopharyngeal cancer)) OR AB=( nasopharyngeal tumor)) OR AB=(nasopharynx tumor)) OR AB=(nasopharynx cancer)) OR AB=(nasopharynx carcinoma)) OR AB=(nasopharynx neoplasms)) OR AB=(NPC)

#2 (((((AB=(metastatic)) OR AB=(Neoplasm Metastasis)) OR AB=(Neoplasm Metastases)) OR AB=(Recurrence)) OR AB=(Neoplasm Recurrence)) OR AB=(Local Neoplasm Recurrence)

#3 ((((((((((((((((AB=(PD-1)) OR AB=(PD-L1))) OR AB=(pembrolizumab)) OR AB=( dostarlimab))) OR AB=(atezolizumab )) OR AB=(avelumab)) OR AB=(durvalumab)) OR AB=( toripalimab)) OR AB=(tislelizumab)) OR AB=(camrelizumab)) OR AB=(cemiplimab)) OR AB=(spartalizumab)) OR AB=( sintilimab)) OR AB=( sugemalimab)) OR AB=(penpulimab)

#1 AND #2 AND #3

**Cochrane library：**

#1 (nasopharyngeal carcinoma):ti,ab,kw OR (nasopharyngeal cancer):ti,ab,kw OR (nasopharyngeal tumor):ti,ab,kw OR (nasopharynx carcinoma):ti,ab,kw OR (NPC):ti,ab,kw

#2 (metastatic):ti,ab,kw OR (Neoplasm Metastasis):ti,ab,kw OR (Neoplasm Metastases):ti,ab,kw OR (Recurrence):ti,ab,kw OR (Neoplasm Recurrence):ti,ab,kw

#3 (pd-1):ti,ab,kw OR (pd-l1):ti,ab,kw

#1 AND #2 AND #3
